# Risk Factors of Postoperative Vomiting in the Eye of “Real-World Evidence”—Modifiable and Clinical Setting-Dependent Risk Factors in Surgical Trauma Patients

**DOI:** 10.3390/jpm11050386

**Published:** 2021-05-08

**Authors:** Yan-Yuen Poon, Ting-Yu Ke, Kuo-Chuan Hung, Hsiao-Feng Lu, Min-Hsien Chiang, Jo-Chi Chin, Shao-Chun Wu

**Affiliations:** 1 Department of Anaesthesiology, Kaohsiung Chang Gung Memorial Hospital, Chang Gung University College of Medicine, No. 123, Ta-Pei Rd., Niao-Song Dist., Kaohsiung City 833, Taiwan; elephant423@gmail.com (Y.-Y.P.); vig529@hotmail.com (T.-Y.K.); maple@cgmh.org.tw (H.-F.L.); ducky0421@gmail.com (M.-H.C.); 2 Department of Anaesthesiology, Chi Mei Medical Center, No. 901, Zhonghua Rd., Yongkang Dist., Tainan City 710, Taiwan; ed102605@gmail.com; 3 Department of Anaesthesiology, Park One International Hospital, No. 100, Bo’ai 2nd Rd., Zuoying Dist., Kaohsiung City 813, Taiwan; jochi731@gmail.com

**Keywords:** postoperative vomiting, risk factors, trauma

## Abstract

Numerous studies on postoperative nausea and vomiting (PONV) have been carried out since the early days of contemporary surgery. The incidence of PONV has been greatly reduced in recent years and new drugs for PONV keep evolving in the market; however, a substantial number of patients are still under the threat of PONV. Female gender, non-smokers, a history of PONV/motion sickness, and postoperative opioid use are four well-recognized risk factors of PONV. Many potential risk factors reported in previous studies were not consistently presented as predictors for PONV. Two questions then arise; are risk factors clinical setting dependent and are risk factors modifiable? We attempted to answer the questions through a comprehensive review of perioperative records of surgical patients from the Trauma Department of our hospital. As nausea is subjective and no standard is applicable for its measurement, postoperative vomiting (POV) was used as an endpoint in this study. To the best of our knowledge, this is the first study to address the POV issue in surgical trauma patients. A total of 855 patients were enrolled in this study after excluding age below 20 years old, total intravenous anesthesia, desflurane anesthesia, or records with missing data. Our results showed that female gender (OR 4.89) is the strongest predicting factor, followed by a less potent predicting factor—more intraoperative opioid consumption (OR 1.07)—which favor more POV. More intraoperative crystalloid supply (OR 0.71) and a higher body weight (OR 0.9) favor less POV. Other potential risk factors did not reach statistical significance in this study as independent risk factors. Our results also showed that when the intraoperative crystalloid infusion rate is greater than 4 mL/kg/h (OR 0.20), it favors a lower rate of POV; when intraoperative opioid consumption is greater than 12 mg morphine equivalents, MME (OR 1.87), it favors a higher rate of POV. We concluded that dominance of any independent risk factor over other risk factors depends on how individual factors interact with the clinical setting. Some risk factors could be modified, and a cut-off value could be derived to facilitate a better plan for POV prevention.

## 1. Introduction

Postoperative nausea and vomiting (PONV) has been one of the obstacles to improving patients’ satisfaction since the early days of modern surgery [1]. Previous reports showed that patients rank the absence of PONV as being important [2] and even rate it as being worse than postoperative pain [3]. An interesting report showed that most patients are willing to pay at their own expense to avoid PONV [4]. With a better understanding of the pathophysiology of PONV [5,6,7,8,9,10] and new drugs [11,12,13,14,15,16,17,18] unceasingly evolving over these years, the incidence of PONV has significantly decreased from 45 to 75% in the early days [19] to 20 to 30% currently [20,21]. 

Epidemiological studies have provided invaluable information of PONV since the early days of surgery and anesthesia. Morphine, ether, cyclopropane, and intraperitoneal surgery were the first discovered factors associated with PONV [22]. The observation of more vomiting in female than male patients was first reported by Davies in 1941 [23]. Now, it is generally accepted that female gender, non-smokers, a history of PONV/motion sickness and postoperative opioids are four independent predictors for PONV, and the Apfel risk scoring system recommends that for patients with at least two out of these four risk factors, necessary precautions to avoid PONV should be considered [24]. There are many effective antiemetic agents available for PONV; their relative risks (RRs) [25] versus placebo for PONV vary between approximately 0.60 and 0.80. Nevertheless, only a fraction of patients gain a quantifiable benefit from them. In fact, PONV is associated with multiple factors, such as the inhalational anesthetics [26,27,28], duration of anesthesia [29,30], perioperative opioids [31,32,33], surgeries [29,30,34,35], intraoperative fluid supply [36], etc. The concept of multimodal therapies for PONV [37] is based on the fact that some PONV-associated factors may be modifiable. It has been reported that dexamethasone [38], antiemetic [39,40], avoidance of dehydration [41,42], total intravenous anesthesia [43], regional anesthesia [44], short-acting opioids [45], and non-steroidal anti-inflammatory drugs (NSAIDs) [46,47] may contribute to PONV reduction, and patients are likely to benefit from risk reduction strategies. However, a substantial number of patients are still under the threat of PONV. Many risk factors reported in previous studies were not consistently presented as predictors for PONV as the four well-recognized risk factors listed above [24]. The present study was accordingly carried out to assess two insufficiently addressed issues. First, are risk factors clinical setting-dependent? Second, could risk factors be modified? Seeking answers to these questions relied on a comprehensive review of pre-, intra-, and post-operative records of surgical patients from the Trauma Department of our hospital.

## 2. Methods

**Figure 1:**
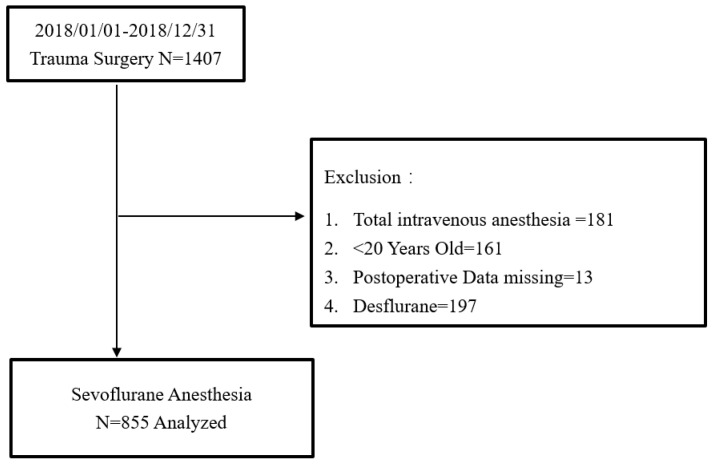
Flow diagram of our surgical trauma patients by sevoflurane-based general anesthesia.

The study was approved by the Institutional Review Board of Kaohsiung Chang Gung Memorial Hospital (IRB number: 202001106B0). Informed consent was waived because of the retrospective nature of the study. All methods were performed in accordance with the relevant guidelines and regulations. A total of 1407 surgical patients from the Trauma Department, from January 2018 to December 2018, were enrolled in the study. Information including medical records, anesthesia records retrieved from the hospital’s electronic database, data during a stay in the post-anesthesia recovery unit (PACU), and data from routine postoperative daily visits were collected. A postoperative visit was performed by well-trained nurse anesthetists within 24 h after surgery. Exclusion criteria included age below 20 years old (the legal threshold of adulthood is 20 in Taiwan), total intravenous anesthesia, desflurane anesthesia, or records with missing data. Finally, 855 patients were included in the study for analyses (Figure 1). 

Nausea is a subjective and unpleasant sensation, of which no standard is applicable for its measurement. Postoperative vomiting (POV) was used as an endpoint and expressed as a dichotomous unit (vomiters or non-vomiters) in the study. POV was defined as vomiting within 24 h of surgery. Individual variables were stratified into three major categories: patient-related variables, anesthesia-related variables, and postoperative course-related variables. Gender, age, body weight, Apfel score, and ASA physical status were assigned to the patient-related category. The type of surgery, duration of anesthesia, sevoflurane consumption, intraoperative fluid supply, red cell transfusion, urine output, intraoperative opioid consumption, use of antiemetic agents and use of antihypertensive agents were assigned to the anesthesia-related category. Opioid consumption at PACU, opioid consumption at ward, and the use of patient-controlled analgesia (PCA) were assigned to the postoperative course-related category.

All general anesthesia were carried out according to the standard procedure suggested by the hospital [48]. Briefly, anesthesia was induced with propofol (1 to 2 mg/kg). Use of rocuronium (1 mg/kg) or cis-atracurium (0.2 mg/kg), fentanyl (1 mcg/kg) or alfentanil (10 mcg/kg), sevoflurane (1 to 1.3 MAC) depended on the anesthesiologists’ preferences. We excluded desflurane anesthesia because of the limited number performed. Sevoflurane concentration was titrated against blood pressure and heart rate changes during anesthesia to maintain stable blood pressure and heart rate within 20% of the patient’s normal range or BIS score was kept in the range of 40 to 60 during anesthesia. A fresh gas flow of 50% oxygen with air was kept at 1 L/min. Maintenance of neuromuscular blocking agents or opioids depended on surgical stimulus, anesthesiologists’ preferences, and objective vital signs. The choice and use of antiemetics were determined by anesthesiologists in charge of the anesthesia. Dexamethasone (5 mg) given at induction or Ondansetron (8 mg) given at 30 min at the end of surgery was the standard antiemetic prescription. 

## 3. Statistical Analysis

Continuous numeric variables were tested by Kolmogorov–Smirnov test for normal distribution. Student’s *t*-test was used to test normally distributed data. Non-normally distributed data were compared using the Mann–Whitney U test and expressed as the median (interquartile range, IQR). The chi-square or Fisher’s exact test was used to analyze categorical variables. Equivalent doses of morphine consumption were not normally distributed and are expressed as the median (IQR) to clearly show the dosage distribution. A univariate analysis and multiple logistic regression model were used to determine the influence of each variable on POV. To investigate the adequate infusion rate of intraoperative crystalloid (mL/kg/h), receiver-operating-characteristic (ROC) and area-under-the-curve analyses were used to determine the best cutoff point for POV prevention based on sensitivity and specificity. Statistical analysis was performed using SPSS (version 22.0; IBM Corp., Armonk, NY, USA). Statistical significance was set at *p* < 0.05.

## 4. Results

A total of 1407 general anesthesia records from January 2018 to December 2018 were retrieved from our hospital’s electronic database. After excluding age below 20 years old, TIVA, desflurane anesthesia, and records with missing data, 855 cases were finally recruited in the study (Figure 1). Table 1 summarizes the demographic characteristics of the patients and the distribution of non-POV and POV patients. In the patient-related category, more female patients developed POV. Younger patients, patients in lower ASA physical status or patients with higher Apfel scores tended to have POV. The average body weight of POV patients was significantly lower than that of non-POV patients. In the anesthesia-related category, sevoflurane consumption (mL/h) or intraoperative opioid consumption (milligram morphine equivalents, MME) was similar between POV and non-POV patients. Intraoperative use of anti-hypertensive drugs, antiemetic, crystalloid fluid supply, blood transfusion, and surgical procedures were similar between POV and non-POV patients. Urine output was significantly less in POV patients. Patients with BIS-guided anesthesia tended to have POV more often than those without BIS. In the postoperative course-related category, opioid consumption at PACU was similar between POV and non-POV patients, while POV patients consumed less opioids at ward.

For quantitative statistical analyses, univariate and multiple logistic regression analyses were performed to explore independent risk factors of POV (Table 2). Among patient-related risk factors, female gender (OR 4.89) was the strongest overall predictor for POV; i.e., the risk of POV in females was 4.89 times higher than in males. Body weight (OR 0.98) was another independent predictor for POV; i.e., when body weight increased by one kilogram, the risk of POV was 0.98 times lower. Age, Apfel score, or ASA physical status were not detected as independent risk factors in our multiple logistic regression model. Among the anesthesia-related risk factors, intraoperative crystalloid supply (OR 0.71) was an independent risk factor; i.e., with a 1 mL/kg/h increase in the crystalloid infusion rate, the risk of POV was 0.71 times lower. Intraoperative opioid consumption (OR 1.07) was another independent risk factor; i.e., when opioid consumption increased by 1 MME, the risk of POV was 1.07 times higher (Table 2). Among the postoperative course-related risk factors, opioid consumption at PACU, opioid consumption at ward, or the use of PCA was not suggested as an independent risk factor (Table 2).

Intraoperative crystalloid supply and intraoperative opioid consumption underwent receiver-operating-characteristic (ROC) analysis in order to gain practical figures for clinical applications. A cut-off value for intraoperative crystalloid supply or intraoperative opioid consumption was determined by the maximal sum of values of specificity and sensitivity (Youden’s index). It implied that when patients had a crystalloid infusion rate greater than 4 mL/kg/h, the risk of POV was one-fifth than the patients who had an infusion rate of 4 mL/kg/h or less (OR 0.20; 0.07-0.56, *p* =0.001). Additionally, it implied that when patients consumed more than 12 MME, the risk of POV was 1.87 times higher than the patients who had 12 MME or less (OR 1.87; 1.15-3.07, *p* =0.011). These two independent risk factors together with gender and body weight were adjusted against surgical types to avoid any interference from surgical procedures and these four independent risk factors remained statistically significant (Table 3 and Supplementary Table S1). 

## 5. Discussion

Studies of postoperative nausea and vomiting have never stopped since the early days of general anesthesia. Thanks to these well-designed clinical trials and corresponding meta-analyses, risk factors of PONV were subsequently revealed and the incidence of PONV was thus greatly reduced. Traditional clinical trials were conducted under well-controlled environments and adhered to a list of eligibility criteria in order to control variability and ensure quality of data [49]. Numerous clinical trials have been carried out to evaluate the effects of inhalational anesthetics [26,27,28], the duration of anesthesia [29,30], type of surgery [34,35], perioperative opioid [31,32,33], perioperative fluid supply [36], etc. on PONV. One of the often-asked questions is, could a small retrospective study of daily performed surgeries reveal risk factors of POV or PONV? It has been reported that for observational studies that involve logistic regression in the analysis, taking a minimum sample size of 500 is necessary to derive the statistics that represent the parameters [50]. Our study included 855 patients, which might be large enough to provide reliable data with a reasonable threshold for good point estimates. It is without a doubt that female gender is a strong predictor for PONV; our results support that female gender is a leading risk factor of POV. One of the interesting findings in this study revealed that young age, patients of low ASA physical status, patients of high Apfel scores, patients with BIS guidance, or patients with less intraoperative urine output were significantly associated with POV in our basic statistical analyses (Table 1). However, these five variables were not detected as independent factors of POV under the scrutiny of multiple logistic regression analyses (Table 2). The discrepancy of these two results (Table 1 and Table 2) may suggest that the occurrence of POV was the consequence of interactions between various pro-vomiting factors in specific underlying clinical settings. Clinical settings of this study were confined to surgeries from the Trauma Department (Table 1), including plastic reconstruction surgeries (14.9%), orthopedic surgeries (2.5%), abdominal surgeries (75.9%), and other general surgery (6.8%). To the best of our knowledge, this is the first study to address the POV issue in surgical trauma patients. Over 80% of anesthesia duration in these surgeries were within 4 h. A review of these abdominal surgeries showed that the procedures were not complicated or time consuming. It is reasonable to speculate that the effects of trauma-induced pain, surgical stimulus and anesthesia-related factors on POV would be less intense under these clinical settings, leaving other risk factors dominant such as female gender, body weight, intraoperative dehydration, and intraoperative opioid consumption (Table 2 and Supplementary Table S1).

In our multiple logistic regression model, four independent risk factors of POV were identified. Female gender as expected was a strong risk factor for POV (OR 4.89). Body weight was another risk factor of POV (OR 0.98); lower body weight favored a higher instance of POV. Generally speaking, females have a lower average body weight than males. Over 52% of patients in this study were female, and this may explain why body weight was identified as an independent risk factor of POV. Intraoperative crystalloid supply was the third independent risk factor of POV in this study. This was in accordance with the results of a previous meta-analysis [36], showing that liberal intraoperative fluid regimens had lower odds of developing PONV as compared with restrictive fluid regimens. However, a recent study [51] showed that a higher occurrence of PONV was found in patients undergoing orthognathic surgery with more intraoperative fluid supply than those with less (≥25 mL/kg vs. <25 mL/kg). Although this study was a 10-year retrospective study, only 101 patients were included, and all factors were considered individually as in our basic statistics (Table 1) which did not detect interactions of different potential risk factors. On the other hand, many studies [36,52,53,54] on intraoperative fluid supply supported more intraoperative fluid supply favored less POV or PONV. Preoperative restrictions on fluid and food intake, together with bowel preparation often cause significant dehydration that may exacerbate POV or PONV. It is justified to consider that intraoperative replenishment of the water deficit to correct hypovolemia may lessen its role on triggering POV or PONV. Meanwhile, a previous study [55] reminded us that liberal fluid administration can lead to fluid overload after recovery from general anesthesia, delayed wound healing, and prolonged hospitalization. Our study derived a cut-off value (> 4 mL/kg/h) for a better management of POV; nevertheless, the mathematics did not tell the safety limits for the infusion rate. Every intraoperative fluid plan should consider patients’ endurance for fluid loading. 

Opioids are effective for many different kinds of pain; however, opioid-induced nausea or vomiting seems to be an unavoidable consequence for many patients [56]. The effects of the perioperative use of opioids on POV or PONV have been widely studied; many support that perioperative opioids favor the occurrence of POV or PONV [24,57,58] in a dose-dependent way [59]. However, most of the studies lack a quantified unit of opioid consumption for comparison; opioid consumption was usually presented as a dichotomous unit [29,34,60,61] (used or not used). For a quantitative comparison of different opioids used in anesthesia, we converted all intraoperative opioids used in this study into a quantified unit, MME for statistical analyses. Our study derived a cut-off value for intraoperative opioid dose (12 MME), suggesting a dose higher than 12 MME-favored POV. However, the mathematics suggested that the lower the dose, the lower the odds of developing POV. Nevertheless, it is not ethical to sacrifice the analgesic effect of opioids for lower odds of POV. Multimodal pain control strategies such as non-opioid analgesic [46,62,63] or regional anesthesia [44] could reduce or eliminate opioid administration and thus reduce the incidence of POV or PONV. Any effective modality for reducing opioid consumption should be considered in the anesthesia planning.

There are a few limitations in our study. First, our study may suffer from potential bias inherent to retrospective studies. Second, the conversion of different opioids into morphine equivalents suffers from an unavoidable bias; the same morphine equivalents of different opioids may have different durations of pro-nausea or pro-vomiting effects. Third, sometimes, nausea is as bad as vomiting for severely bothering postoperative patients; however, an evaluation of its severity in detail or occurrence was not available in this study. A well-designed questionnaire for POV is required and warranted for our future study of POV. Fourth, PON and PONV are important complications of general anesthesia but they were not included for analysis. Finally, we concluded that first, any independent risk factor of POV identified is the consequence of the interactions of various potential risk factors under specific clinical settings. Second, a cut-off value derived from modifiable risk factors may help those highly susceptible patients for POV. Third, independent risk factors are clinical setting-dependent, setting up guidelines of POV prevention for surgeries with similar clinical settings may improve the overall patients’ satisfaction. Fourth, the main result was based on deductions from the “real-world” data, so further prospective studies are necessary to verify our results, especially the two derived cut-off values.

## Figures and Tables

**Table 1:** Demographic and clinical characteristic features of surgical trauma patients.

Features	N (%)/Median (IQR)	None-POV	POV	*p* Value
**Gender**				
Male	404 (47.3%)	381 (51.0%)	23 (21.3%)	<0.001
Female	451 (52.7%)	366 (49.0%)	85 (78.7%)	
**Age** (years)				
20–49	322 (37.7%)	270 (36.1%)	52 (48.1%)	0.030
50–69	382 (44.7%)	338 (45.2%)	44 (40.7%)	
70 and above	151 (17.7%)	139 (18.6%)	12 (11.1%)	
**Weight** (kg)	65.0 (57.0–75.0)	66.0 (58.0–75.0)	62.0 (54.0–70.0)	0.004
**BIS**				
None	471 (55.1%)	423 (56.6%)	48 (44.4%)	0.017
Yes	384 (44.9%)	324 (43.4%)	60 (55.6%)	
**Sevoflurane consumption** (mL/h)	11.43 (9.20–13.73)	11.43 (9.16–13.85)	11.64 (9.51–13.27)	0.915
**Intraoperative MME** (mg)	13.0 (10.0–180)	13.0 (10.0–18.0)	13.0 (13.0–18.0)	0.334
**MME at PACU** (mg)	0 (0–0)	0 (0–0)	0 (0–0)	0.379
**MME at WARD** (mg)	0 (0–3.0)	0 (0–3.5)	0 (0–0)	0.045
**ASA**				
Ⅰ	47 (5.5%)	39 (5.2%)	8 (7.4%)	0.002
Ⅱ	575 (67.3%)	489 (65.5%)	86 (79.6%)	
Ⅲ	233 (27.3%)	219 (29.3%)	14 (13.0%)	
**Anesthesia Time** (hour)	2.7 (2.2–3.6)	2.8 (2.2–3.6)	2.5 (2.2–3.3)	0.240
**Anesthesia Time** (hour)				
<2	171 (20.0%)	152 (20.3%)	19 (17.6%)	0.205
2- < 4	517 (60.5%)	444 (59.4%)	73 (67.6%)	
4- < 6	107 (12.5%)	94 (12.6%)	13 (12.0%)	
6 and above	60 (7.0%)	57 (7.6%)	3 (2.8%)	
**Apfel Score**				
0	143 (20.2%)	134 (21.6%)	9 (10.2%)	0.003
1	295 (41.6%)	263 (42.4%)	32 (36.4%)	
2 and above	271 (38.3%)	224 (36.1%)	47 (53.4%)	
**Comorbidity Index**				
0	509 (59.5%)	432 (57.8%)	77 (71.3%)	0.007
1	177 (20.7%)	155 (20.7%)	22 (20.4%)	
2	88 (10.3%)	81 (10.8%)	7 (6.5%)	
≥3	81 (9.5%)	79 (10.6%)	2 (1.9%)	
**Kinds of Antiemetic Drugs**				
None	483 (56.5%)	420 (56.2%)	63 (58.3%)	0.418
One	315 (36.8%)	274 (36.7%)	41 (38.0%)	
Two and above	57 (6.7%)	53 (7.1%)	4 (3.7%)	
**Crystalloid** (mL/h/Kg)	2.49 (1.91–3.32)	2.52 (1.93–3.35)	2.38 (1.79–3.06)	0.081
**Intraoperative Urine** (mL/h/Kg)	13.18 (10.10–16.25)	14.08 (10.65–17.51)	6.95 (1.49–12.42)	0.043
**Red Blood Transfusion** (mL/kg/h)	36.56 (13.48–59.63)	41.17 (14.80–67.54)	4.63 (4.55–13.81)	0.169
**Kinds of Intraoperative anti-hypertensive drugs**				
None	570 (66.7%)	496 (66.4%)	74 (68.5%)	0.812
One	219 (25.6%)	194 (26.0%)	25 (23.1%)	
Two and above	66 (7.7%)	57 (7.6%)	9 (8.3%)	
**Surgical Type**				
Plastic Reconstructive	127 (14.9%)	116 (15.5%)	11 (10.2%)	0.136
Orthopedic	21 (2.5%)	20 (2.7%)	1 (0.9%)	
General surgery except abdomen	58 (6.8%)	53 (7.1%)	5 (4.6%)	
Abdominal surgery (including hepatobiliary, spleen and GI tract)	649 (75.9%)	558 (74.7%)	91 (84.3%)	
**Patient Controlled Analgesia**				
None	793 (92.7%)	690 (92.4%)	103 (95.4%)	0.261
Yes	62 (7.3%)	57 (7.6%)	5 (4.6%)	

**Table 2:** Univariate and multiple logistic regression model of postoperative vomiting.

Variables (Unit)	N (%)	Univariate		Multivariable	
		OR (95% CI)	*p* Value	OR (95% CI)	*p* Value
**Gender**-Male	404 (47.3%)	1		1	
Gender-Female	451 (52.7%)	3.85 (2.37–6.23)	<0.001	4.89 (1.91–12.50)	0.001
**Age**-20–49	322 (37.7%)	1		1	
Age-50–69	382 (44.7%)	0.68 (0.44–1.04)	0.076	0.56 (0.27–1.18)	0.128
Age-70 and above	151 (17.7%)	0.45 (0.23–0.87)	0.017	0.56 (0.20–1.53)	0.255
**Weight** (kg)	855 (100.0%)	0.98 (0.96–0.99)	0.009	0.98 (0.95–1.00)	0.044
**BIS**-none	471 (55.1%)	1		1	
BIS-Yes	384 (44.9%)	1.63 (1.09–2.45)	0.018	1.47 (0.87–2.46)	0.147
**Apfel Score** 0	143 (20.2%)	1		1	
Apfel Score 1	295 (41.6%)	1.81 (0.84–3.91)	0.130	0.57 (0.20–1.66)	0.305
Apfel Score 2	216 (30.5%)	2.78 (1.29–5.99)	0.009	0.43 (0.11–1.76)	0.243
Apfel Score 3&4	55 (7.8%)	4.61 (1.84–11.54)	0.001	0.53 (0.11–2.63)	0.441
**ASA** Ⅰ	47 (5.5%)	1		1	
ASA Ⅱ	575 (67.3%)	0.86 (0.39–1.90)	0.704	0.79 (0.30–2.10)	0.637
ASA Ⅲ	233 (27.3%)	0.31 (0.12–0.79)	0.014	0.51 (0.15–1.68)	0.267
**Sevoflurane consumption** (mL/h)	855 (100.0%)	0.98 (0.93–1.03)	0.349	1.00 (0.95–1.06)	0.890
**Duration** < 2 (hour)	171 (20.0%)	1		1	
−2–4	517 (60.5%)	1.32 (0.77–2.25)	0.317	1.30 (0.67–2.51)	0.441
−4–6	107 (12.5%)	1.11 (0.52–2.34)	0.792	1.34 (0.53–3.39)	0.533
≥6 and above	60 (7.0%)	0.42 (0.12–1.48)	0.177	0.98 (0.19–4.95)	0.981
**Crystalloid** (mL/h/Kg)	855 (100.0%)	0.81 (0.68–0.95)	0.010	0.71 (0.55–0.92)	0.009
**Red Blood Transfusion** (mL/h/Kg)	855 (100.0%)	1.00 (1.00–1.00)	0.259	1.00 (1.00–1.00)	0.892
**Intraoperative Urine** (mL/h/Kg)	855 (100.0%)	0.99 (0.99–1.00)	0.140	1.00 (0.99–1.01)	0.792
**Intraoperative MME** (mg)	855 (100.0%)	1.01 (0.98–1.05)	0.439	1.07 (1.01–1.13)	0.016
**MME at PACU** (mg)	855 (100.0%)	1.06 (0.92–1.23)	0.405	0.98 (0.81–1.17)	0.797
**MME at WARD** (mg)	855 (100.0%)	0.95 (0.90–1.00)	0.056	0.96 (0.90–1.02)	0.184
**PCA**-none	793 (92.7%)	1		1	
PCA-Yes	62 (7.3%)	0.59 (0.23–1.50)	0.266	1.10 (0.30–3.98)	0.883
**Kinds of anti-emetics**-none	483 (56.5%)	1		1	
One	315 (36.8%)	1.00 (0.65–1.52)	0.991	0.67 (0.40–1.12)	0.125
Two	57 (6.7%)	0.50 (0.18–1.44)	0.200	0.39 (0.10–1.57)	0.185
**Kinds of anti-hypertension**-none	570 (66.7%)	1		1	
One	219 (25.6%)	0.86 (0.53–1.40)	0.552	0.97 (0.53–1.75)	0.910
Two	66 (7.7%)	1.06 (0.50–2.23)	0.881	0.98 (0.39–2.45)	0.965

**Table 3:** Multiple logistic regression model of postoperative vomiting adjusted by surgical type.

Model	Crystalloid (mL/kg/h)	*p*	Intraoperative MME (mg)	*p* Value	Female (Yes/No)	*p* Value	Weight (kg)	*p* Value
Main model (Table 2)	0.71 (0.55–0.92)	0.009	1.07 (1.01–1.13)	0.016	4.89 (1.91–12.50)	0.001	0.98 (0.95–1.00)	0.044
Adjusted by surgical type (Supplementary Table S1)	0.67 (0.52–0.88)	0.003	1.06 (1.00–1.12)	0.035	4.98 (1.93–12.82)	0.001	0.97 (0.95–1.00)	0.023

## Supplementary Materials

The following are available online at https://www.mdpi.com/article/10.3390/jpm11050386/s1, Table S1: Multiple logistic regression model of postoperative vomiting adjusted by surgical type.
