# Supplementary material for: Risk Factors of Postoperative Vomiting in the Eye of “Real-World Evidence”—Modifiable and Clinical Setting-Dependent Risk Factors in Surgical Trauma Patients
Source: J Pers Med. 2021 May 8;11(5):386. doi: 10.3390/jpm11050386 (PMC8151314; doi:10.3390/jpm11050386)
Supplement: Supplementary file 1 [file jpm-11-00386-s001.zip › jpm-1183379-supplementary.pdf]

Supplemental Table S1. Multiple logistic regression model of postoperative vomiting adjusted by surgical type.

Variables (Unit)	N(%)	Univariate		Multivariable	
		OR (95% CI)	*p*-Value	OR (95% CI)	*p* Value
**Gender-Male**	404 (47.3%)	1		1	
Gender-Female	451 (52.7%)	3.85 (2.37–6.23)	<0.001	4.98 (1.93–12.82)	0.001
**Age**-20–49	322 (37.7%)	1		1	
Age-50–69	382 (44.7%)	0.68 (0.44–1.04)	0.076	0.53 (0.25–1.12)	0.098
Age-70 and above	151 (17.7%)	0.45 (0.23–0.87)	0.017	0.50 (0.18–1.38)	0.184
**Weight** (kg)	855 (100.0%)	0.98 (0.96–0.99)	0.009	0.97 (0.95–1.00)	0.023
**BIS**-none	471 (55.1%)	1		1	
BIS-Yes	384 (44.9%)	1.63 (1.09–2.45)	0.018	1.28 (0.74–2.20)	0.374
**Apfel Score** 0	143 (20.2%)	1		1	
Apfel Score 1	295 (41.6%)	1.81 (0.84–3.91)	0.130	0.57 (0.20–1.68)	0.312
Apfel Score 2	216 (30.5%)	2.78 (1.29–5.99)	0.009	0.43 (0.10–1.79)	0.248
Apfel Score 3&4	55 (7.8%)	4.61 (1.84–11.54)	0.001	0.55 (0.11–2.76)	0.467
**ASA** I	47 (5.5%)	1		1	
ASA II	575 (67.3%)	0.86 (0.39–1.90)	0.704	0.75 (0.28–2.04)	0.576
ASA III	233 (27.3%)	0.31 (0.12–0.79)	0.014	0.45 (0.13–1.53)	0.203
**Sevoflurane consumption** (mL/hr)	855 (100.0%)	0.98 (0.93–1.03)	0.349	1.00 (0.95–1.06)	0.927
**Duration** < 2 (hour)	171 (20.0%)	1		1	
−2–4	517 (60.5%)	1.32 (0.77–2.25)	0.317	1.02 (0.51–2.02)	0.966
−4–6	107 (12.5%)	1.11 (0.52–2.34)	0.792	1.44 (0.55–3.75)	0.456
≥6 and above	60 (7.0%)	0.42 (0.12–1.48)	0.177	1.07 (0.21–5.47)	0.936
**Crystalloid** (mL/hr/Kg)	855 (100.0%)	0.81 (0.68–0.95)	0.010	0.67 (0.52–0.88)	0.003
**Red Blood Transfusion** (mL/hr/Kg)	855 (100.0%)	1.00 (1.00–1.00)	0.259	1.00 (1.00–1.00)	0.801
**Intraoperative Urine** (mL/hr/Kg)	855 (100.0%)	0.99 (0.99–1.00)	0.140	1.00 (0.99–1.01)	0.618
**Intraoperative MME** (mg)	855 (100.0%)	1.01 (0.98–1.05)	0.439	1.06 (1.00–1.12)	0.035
**MME at PACU** (mg)	855 (100.0%)	1.06 (0.92–1.23)	0.405	0.98 (0.81–1.18)	0.832
**MME at Ward** (mg)	855 (100.0%)	0.95 (0.90–1.00)	0.056	0.97 (0.91–1.03)	0.323
**PCA-none**	793 (92.7%)	1		1	
PCA-Yes	62 (7.3%)	0.59 (0.23–1.50)	0.266	0.94 (0.26–3.42)	0.919
**Kinds of anti-emetics**-none	483 (56.5%)	1		1	
One	315 (36.8%)	1.00 (0.65–1.52)	0.991	0.67 (0.40–1.12)	0.130
Two	57 (6.7%)	0.50 (0.18–1.44)	0.200	0.42 (0.10–1.69)	0.220
**Kinds of anti-hypertension**-none	570 (66.7%)	1		1	
One	219 (25.6%)	0.86 (0.53–1.40)	0.552	1.05 (0.58–1.93)	0.864
Two	66 (7.7%)	1.06 (0.50–2.23)	0.881	1.24 (0.48–3.19)	0.655
**Surgical type**					
Plastic Reconstructive	127 (14.9%)	1		1	
Orthopedic	21 (2.5%)	0.53 (0.06–4.31)	0.551	0.52 (0.06–4.66)	0.556
General surgery except abdomen	58 (6.8%)	1.00 (0.33–3.01)	0.993	0.63 (0.17–2.30)	0.480
Abdominal surgery (including hepatobiliary, spleen and GI tract)	649 (75.9%)	1.72 (0.89–3.32)	0.106	1.96 (0.83–4.65)	0.124
